# Evaluation of Clinical Contributions Provided by Addition of the Brain, Calvarium, and Scalp to the Limited Whole Body Imaging Area in FDG-PET/CT Tumor Imaging

**DOI:** 10.1155/2014/129683

**Published:** 2014-06-16

**Authors:** Bekir Tasdemir, Zeki Dostbil, Ali Inal, Kemal Unal, Sule Yildirim, F. Selcuk Simsek

**Affiliations:** ^1^Department of Nuclear Medicine, Faculty of Medicine, Dicle University, 21280 Diyarbakir, Turkey; ^2^Department of Medical Oncology, Faculty of Medicine, Dicle University, 21280 Diyarbakir, Turkey; ^3^Department of Nuclear Medicine, Faculty of Medicine, Izmir University, 35575 Izmir, Turkey; ^4^Department of Nuclear Medicine, Elazig Training and Research Hospital, 23200 Elazig, Turkey

## Abstract

*Purpose.* The aim of this study was to detect additional findings in whole body FDG-PET/CT scan including the brain, calvarium, and scalp (compared to starting from the base of the skull) in cancer patients and to determine contributions of these results to tumor staging and treatment protocols. *Materials and Methods.* We noted whether the findings related to the brain, calvarium, and scalp in 1359 patients had a potential to modify staging of the disease, chemotherapy protocol, radiotherapy protocol, and surgical management. We identified rates of metastatic findings on the brain, calvarium, and scalp according to the tumor types on FDG-PET/CT scanning. *Results.* We found FDG-PET/CT findings for malignancy above the base of the skull in 42 patients (3.1%), one of whom was a patient with an unknown primary tumor. Twenty-two of the metastatic findings were in the brain, 16 were in the calvarium, and two were in the scalp. *Conclusion.* This study has demonstrated that addition of the brain to the limited whole body FDG-PET/CT scanning may provide important contributions to the patient's clinical management especially in patients with lung cancer, bladder cancer, malignant melanoma, breast cancer, stomach cancer, and unknown primary tumor.

## 1. Introduction

Positron emission tomography (PET) is a tomographic scintigraphic imaging technique which detects the annihilation photons that are emitted from radionuclides which decay and release positrons [1]. Combination of the computed tomography (CT) provides attenuation correction and anatomical information to the PET imaging. F-18 2-fluoro-2-deoxy-D-glucose (FDG) is a radiolabeled analog of glucose which is used for PET/CT imaging. FDG is transported through the cell membrane by glucose transport proteins (GLUTs) and phosphorylated by hexokinase [2]. FDG-PET imaging is a noninvasive imaging technique that shows the glucose uptake of the cells in the body [3]. Metabolic imaging using FDG-PET plays crucial role in management of the cancer patients.Currently, PET/CT whole body imaging with FDG is an accepted imaging method which is widely used for staging, restaging, and treatment monitoring of several cancers. Although FDG-PET/CT whole body imaging is considered to be a whole body scan, in clinical practice, it usually includes the body area limited to the distance between the base of the skull and the proximal thigh. This type of scanning is known as limited whole body imaging [4]. Although limited whole body imaging is an accepted practical application and has been entered into the guidelines, adding other body parts to the limited whole body imaging area is recommended for tumors that have a high probability of involvement of the scalp, calvarium, skull, brain, or lower extremities.

As there is no clear statement in the guidelines for the field of view (FOV) in FDG-PET/CT tumor imaging [1, 5–7], different practices are administered in different clinics. Huston et al. drew attention to this issue in their published article where they emphasized that there was a need for a standardized FOV [7]. Some recent studies evaluating inclusion of the rest of the parts of the body to the limited whole body FOV have been published, but new studies performed on large patient populations are still needed [4, 8–15].

The aim of this study was to detect additional findings in whole body FDG-PET/CT scan including the brain, calvarium, and scalp (compared to starting from the base of the skull) in cancer patients and to determine contributions of these results to tumor staging and treatment protocols.

## 2. Materials and Methods

### 2.1. Patient Population

We performed this study retrospectively on medical charts of 1359 patients from Elazığ Education and Research Hospital that were dated from January 2010 to August 2012. Demographic data were collected from the chart records. All patients had been diagnosed with cancer. The patients had been referred to the Nuclear Medicine Department for FDG-PET/CT scanning for tumor imaging. Images of all patients had been evaluated and reported by the authors. Patients with primary tumors of the brain, scalp, and calvarium, as well as those having repeated PET/CT scans in the past year, were excluded from the study.

### 2.2. Imaging

Patients were advised to limit their physical activity on the day prior to the examination. Patients fasted for a minimum of four hours prior to imaging. Patients' blood glucose levels were measured before FDG injection. FDG was injected intravenously at a mean dose of 370 MBq/70 kg body weight. Patients were also kept as still as possible in a quiet room during FDG injection and during the postinjection waiting period of 60 minutes and advised to be quiet. Biograph 6 PET/CT scanner (Siemens Medical Systems, Knoxville, TN) was used for the imaging procedure. First CT scan and then PET scan were performed. Although they vary according to the height of the patients, CT and PET images were obtained at an average of seven bed positions (from vertex to proximal thigh). Imaging began with the head. The arms were placed down below the navel during the imaging of head and neck tumors and above the head during the imaging of other types of tumors. The CT component of the integrated scanner was 6-slice. Energy level of the CT imaging was 130 KV and 80 mAs. Slice thickness was set to 6 mm. The detector crystal in the PET scanner was LSO. PET emission scans were acquired for 3 min/bed position in 128 × 128 matrix. TrueD software program was used to evaluate the images.

### 2.3. Image Evaluation and Analysis

**Figure 1:**
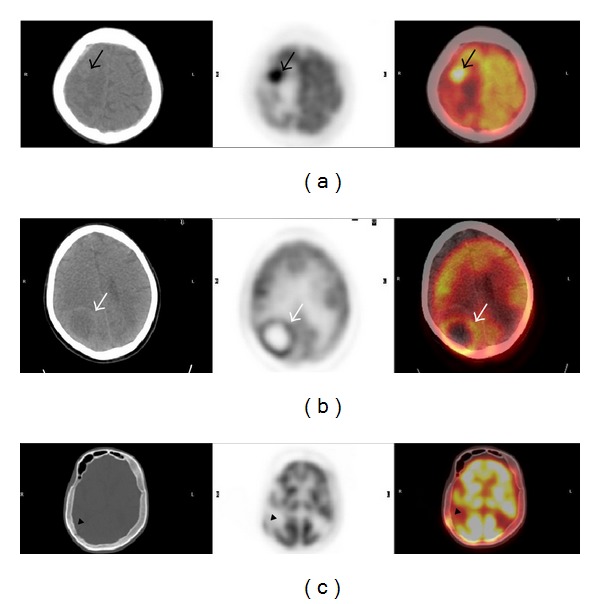
FDG-PET/CT images of several oncological patients demonstrating findings for metastasis. (a) shows transverse unenhanced CT, PET, and PET/CT fusion images of a 64-year-old man with primary unknown tumor. Focal hypermetabolic area (black arrows) is consistent with a brain metastasis. (b) shows transverse unenhanced CT, PET, and PET/CT fusion images of a 65-year-old man with lung cancer. Peripherally hypermetabolic and centrally hypometabolic area (due to a necrotic tissue) (white arrows) is consistent with a brain metastasis. (c) shows transverse unenhanced CT, PET, and PET/CT fusion images of a 27-year-old man with non-Hodgkin lymphoma. Focal hypermetabolic area (black arrow heads) is consistent with a calvarial bone metastasis.

We routinely use vertex to proximal thigh imaging in our clinic. Therefore, the brain, scalp, and calvarium were within the scanning area in all patients. We regarded high FDG uptake relative to the background (adjacent tissues) as a malignant or metastatic finding on FDG-PET/CT (Figure 1), and we checked data of all patients and noted malignant or metastatic findings on the brain, the calvarium, and the scalp. We then consulted with a medical oncologist concerning these findings with regard to tumor types. We noted whether the findings related to the brain, calvarium, and scalp had a potential to modify staging of the disease, chemotherapy protocol, radiotherapy protocol, and surgical management. Then, we identified rates of metastatic findings on the brain, calvarium, and scalp according to the tumor types on FDG-PET/CT scanning.

## 3. Results

Of a total of 1359 patients, 645 were women (47.5%) and 714 were men (52.5%), and the mean age was 57 ± 15.6 years. Blood glucose levels in eight patients were between 200 and 226 mg/dL and were below 200 mg/dL in the rest. We found malignant findings above the base of the skull in 42 patients (3.1%), one of whom was a patient with an unknown primary tumor. Twenty-four metastatic findings were in the brain, sixteen were in the calvarium, and two were in the scalp. Tumor types and the number of patients are shown in Table 1, and distribution of the metastatic findings detected in brain, calvarium, and scalp according to tumor types is shown in Table 2. There were no patients who had metastatic findings in more than one area.

Metastatic findings in the brain were detected most frequently in patients with lung cancer. Four of the 24 metastatic findings had a feature that could change the stage of the disease, five had a feature that could change the chemotherapy protocol, all of them had a feature that could change the radiotherapy protocol, and one of them had a feature that could change surgical management (Table 3).

Metastatic findings in the calvarium and scalp did not have any features that could change the staging of the disease, chemotherapy protocol, radiotherapy protocol, or surgical management. However, one malignant finding in the scalp of one patient had a property that could be the primary focus of an unknown primary tumor.

Under the “other tumors” category in Table 2, one metastatic finding in the calvarium was seen in a patient with ocular adenoid cystic cancer and one metastatic finding in the brain was seen in a patient with ileum cancer.

## 4. Discussion

Recently, several studies assessing the addition of part of or all of other body regions to limited whole body PET/CT scanning have been published [4, 8–15]. To add the rest of other body regions to the limited whole body PET/CT scanning may provide more comprehensive data to clinicians. However, the contribution of every part of the body region to be added to the limited whole body PET/CT scanning is not equally valuable clinically. For example, Sebro et al. have stated in their study that the clinical impact of PET/CT findings in the brain was more prominent than in lower extremities (1.1% and 0.2%, resp.) [11]. Taking into account these findings, it is more appropriate to discuss the addition of the brain to the typical limited whole body FOV (base of skull to upper thigh) than the other regions. Thus, in this study we assessed to what extent the metastatic findings identified in the brain provided clinical contributions. In this study, we had an opportunity to evaluate the clinical contributions of metastatic findings of the calvarium and scalp because they were also included in the FOV in addition to the brain.

In the present study, we identified brain, calvarium, and scalp lesions in 42 of 1359 patients (3.1%) on PET/CT. In their study, Abdelmalik et al. have reported that there were substantial FDG-PET/CT findings above the base of the skull in 10.2% of their patients [8]. This rate may be higher than the actual value because, in addition to malignancies, they also included some benign lesions. Thus, they expressed their findings as substantial FDG-PET/CT lesions rather than malignancies [8].

In this study, 24 of the total metastatic findings detected in the brain, calvarium, and scalp were located in the brain (1.8%). This is consistent with findings of other studies in which reported ranges were between 1% and 1.6% [4, 11, 12]. In fact, the incidence of brain metastasis in all types of tumors is not known. However, in the literature, the rates reported ranged between 8.5% and 9.6% for tumors such as lung cancer, colon cancer, breast cancer, renal cell cancer, and malignant melanoma that frequently involve brain metastases in comprehensive oncological studies [16–18]. Because our study, together with similar ones performed using FDG-PET/CT, included many different tumor types other than those known to involve frequent brain metastases, the detected brain metastasis ratios were markedly higher. Furthermore, the efficiency of FDG-PET/CT to detect brain metastases is low because the grey matter of the brain is a hypermetabolic tissue. As a result of this hypermetabolism, brain metastasis ratios have generally been low in PET/CT studies [6, 14].

In our study, we detected lesions consistent with brain metastases most frequently in lung cancer (5.33%). This rate is relatively higher than similar studies in which reported values ranged between 1.5% and 4.9% [4, 9, 11]. In the present study, because the patients with suspected lung cancer who underwent diagnostic FDG-PET/CT scanning were classified into a separate group, our rates may have been a bit higher. Thus, if patients with lung cancer and those with suspected lung cancer were taken together into the same group, the ratio would have been 3.1%, which is consistent with that of prior studies. We assessed patients with lung cancer and with suspected lung cancer in separate groups. A significant difference between rates of brain metastasis in these two groups was observed (5.33% and 1.7%, resp.). This demonstrates that one should be more careful with patients with lung cancer than with those with suspected lung cancer in terms of detection of brain metastases. However, in patients with suspected lung cancer, it is beneficial to be careful for brain metastases. For example, in a patient with suspected lung cancer, we detected a lesion consistent with brain metastasis that had potential to change the stage and surgical treatment planning. If the brain region of this patient had not been taken into scanning area, the lesion consistent with brain metastasis would not have been detected and perhaps unnecessary surgical intervention would have taken place in this case.

In our PET/CT study we found bladder cancer (5.26%) was the second most frequent cause of brain metastases after lung cancer (5.33%). This rate is very close to the rate of 5.9% reported by Sebro et al. for brain metastases [11]. However, in some other FDG-PET/CT studies, brain metastases were not detected [4, 8, 12].

In our study, malignant melanoma and breast cancer were two other common types of cancers in which the brain metastases were most frequently detected (3.1% and 2.5%, resp.), rounding out the five tumor types that were the most frequent cause of the brain metastases (Table 1) [16–18]. Bochev et al. reported in a similar FDG-PET/CT study that the brain metastasis rate was 3.4% for malignant melanoma and 0.8% for breast cancer [12]. Their value for malignant melanoma (3.4%) is quite close to ours (3.1%) [12]. However, in some similar studies, no brain metastases were detected [4, 8]. Also, Sebro et al. reported that the brain metastasis rate for malignant melanoma was 7.1%, and they did not detect any brain metastases in patients with breast cancer [11]. Although it has been reported in the literature that renal cell cancer and colorectal cancer frequently cause brain metastases, we did not detect any FDG-PET/CT findings consistent with the brain metastasis [16–18]. In some similar FDG-PET/CT studies, rates of brain metastasis were reported to be 0%–11.1% for renal cell cancer and 0%–0.7% for colorectal cancer [4, 8, 11, 12].

Another conspicuous finding in our study was extra metastatic lesions in the calvarium that were detected in sixteen patients and extra metastatic lesions in the scalp detected in two patients which did not provide any additional contribution to the patient's management.

However, in a patient with an unknown primary tumor we detected an FDG-PET/CT finding consistent with the primary origin in scalp. Additionally, addition of part of other body regions to limited whole body PET/CT scan may reveal extra findings. However, the number of additional abnormal findings detected and their importance for clinical management are the main concerns. Furthermore, extra radiation exposure due to the CT portion of the imaging procedure for additional body parts, extra time spent, and an increase in the cost due to increased need for FDG in conjunction with the prolonged scanning time should be considered. That is, the work to be done should be reasonable and cost-effective. The brain is a relatively radiation-resistant organ, and it can be imaged at one bed position. Moreover, detection of a metastasis in the central nervous system, especially in the early period, may give an opportunity for clinicians to prevent many serious neurological complications.

Results of this study are based on the analysis of FDG-PET/CT scans of a large number of patients as a whole. But number of patients is small in some tumor types, especially in the mesothelioma, basal cell cancer, parotid cancer, pancreatic cancer, testicular cancer, thyroid cancer, and bladder cancer. Therefore, the ratio of metastatic findings may vary in these tumor types in larger patient population.

In conclusion, the findings of this study have demonstrated that addition of the brain to the limited whole body FDG-PET/CT scanning may provide important contributions to the patient's clinical management in patients with lung cancer, bladder cancer, malignant melanoma, breast cancer, stomach cancer, and unknown primary tumor. On this issue, more comprehensive studies should be performed with patients having these types of tumors to get more detailed information.

## Figures and Tables

**Table 1:** Tumor types and number of patients in the study.

Diagnosis	Number of patients
Lung Ca	150
Lung Ca?	240
Breast Ca	160
Unknown primary Tm	95
Non-Hodgkin lymphoma	107
Bladder Ca	19
Malignant melanoma	32
Gastric Ca	46
Thyroid Ca	11
Hodgkin lymphoma	78
Esophageal Ca	12
Colon Ca	35
Rectal Ca	28
Uterine Ca	36
Over Ca	36
Cervix Ca	18
Renal cell Ca/Wilms' Tm	25
Prostate Ca	21
Testicular Ca	12
Pancreatic Ca	11
Pancreatic Ca?	13
Mesothelioma	3
Mesothelioma?	25
Squamous cell Ca	18
Basal cell Ca	9
Sarcomas	13
Nasopharynx Ca	12
Parotid Ca	9
Other head and neck Tm	21
Other tumors	64

Total	1359

Ca: cancer, Tm: tumor, ?: suspected.

**Table 2:** Distribution of the metastatic findings detected in brain, calvarium, and scalp according to tumor types.

Diagnosis	Number of patients	Brain Met		Calvarium Met		Scalp Met		Total	
		*n*	%	*n*	%	*n*	%	*n*	%
Lung Ca	150	8	*5.33 *	2	*1.3 *	1	*0.7 *	11	*7.3 *
Lung Ca?	240	4	*1.7 *	1	*0.4 *	—	—	5	*2.1 *
Breast Ca	160	4	*2.5 *	5	*3.1 *	1	*0.6 *	10	*6.2 *
Unknown primary Tm	95	3	*3.2 *	2	*2.1 *	—	—	5	*5.3 *
NHL	107	1	*0.9 *	3	*2.8 *	—	—	4	*3.7 *
Bladder Ca	19	1	*5.26 *	1	*5.3 *	—	—	2	*10.5 *
Malignant melanoma	32	1	*3.1 *	—	—	—	—	1	*3.1 *
Gastric Ca	46	1	*2.2 *	—	—	—	—	1	*2.2 *
Thyroid Ca	11	—	—	1	*9.1 *	—	—	1	*9.1 *
Other tumors	499	1	*0.2 *	1	*0.2 *	—	—	2	*0.4 *

Total	1359	24	*1.8 *	16	*1.2 *	2	*0.1 *	42	*3.1 *

Ca: cancer, Tm: tumor, Met: metastasis, *n*: number, ?: suspected, NHL: Non-Hodgkin lymphoma.

**Table 3:** Clinical effects of the metastatic findings detected in brain on FDG-PET/CT scanning.

Diagnosis	Brain Met	Effect on stage	Effect on RT	Effect on CHT	Effect on surgery
Lung Ca	8	1	8	—	—
Lung Ca?	4	1	4	1	1
Breast Ca	4	—	4	2	—
Primary unknown Tm	3	—	3	—	—
Non-Hodgkin lymphoma	1	1	1	1	—
Bladder Ca	1	1	1	1	—
Malignant melanoma	1	—	1	—	—
Gastric Ca	1	—	1	—	—
Other tumors	1	—	1	—	—

Total	24	4	24	5	1

Ca: cancer, Tm: tumor, Met: metastasis, RT: radiotherapy, ?: suspected, CHT: chemotherapy.
